# A developmental outlook on the role of cognition and emotions in youth volleyball and artistic gymnastics

**DOI:** 10.3389/fpsyg.2022.954820

**Published:** 2022-08-10

**Authors:** Elisa Bisagno, Alessia Cadamuro, Sandro Rubichi, Claudio Robazza, Francesca Vitali

**Affiliations:** ^1^Department of Law, University of Modena and Reggio Emilia, Modena, Italy; ^2^Department of Biomedical, Metabolic and Neural Sciences, University of Modena and Reggio Emilia, Modena, Italy; ^3^Department of Medicine and Aging Sciences, Behavioral Imaging and Neural Dynamics Center, G. d’Annunzio University of Chieti and Pescara, Chieti, Italy; ^4^Section of Movement Sciences, Department of Neurosciences, Biomedicine and Movement Sciences, University of Verona, Verona, Italy

**Keywords:** working memory, executive functions, attentional style, emotions, youth sport, cognition

## Abstract

Developmental and cognitive psychology recently started to take an interest in the sports domain, exploring the role of either cognitive functions or emotions in youth sport. However, to the extent that cognition and emotions are inextricably linked, studying them jointly from a developmental perspective could inform on their interplay in determining performance in different sports. This research examined the role of general cognitive abilities, attentional style, and emotions (controlling for age and experience), in predicting performance in youth volleyball and artistic gymnastics. A total of 218 female participants, of which 114 volleyball players and 104 artistic gymnasts (11–17 years old) were administered two measures of working memory and six measures of executive functions (namely inhibition, updating, and shifting). They also completed an attentional style and an emotion-related questionnaire. For each volleyball player, an individual performance index based on every gesture performed during the games and controlled for the team performance was computed. As a measure of gymnasts’ performance, scores in 2017–2018 competitions were used. Regression analysis showed that the main predictor of the volleyball players’ performance (R^2^ = 0.23) was a working memory-updating factor (ß = 0.45, *p* = 0.001), together with experience (ß = 0.29, *p* = 0.030) and high-arousal unpleasant emotions (ß = 0.30, *p* = 0.029), which positively predicted performance. Experience (ß = 0.30, *p* = 0.011), age (ß = −0.036, *p* = 0.005) and high-arousal unpleasant emotions (ß = −0.27, *p* = 0.030) were the predictors of gymnasts’ performance (R^2^ = 0.25). These results represent a first step in understanding if and how youth female athletes of open- and closed-skills sports rely on different psychological abilities. This line of research could offer insight to practitioners regarding which psychological abilities could be more relevant to train depending on the type of sport.

## Introduction

In the last decade, there has been growing research interest in the role of cognitive functioning in determining sports performance (e.g., Vaughan and Laborde, 2021). However, the relationship between cognition and performance of youth athletes has been almost overlooked [for an exception, see Ishihara et al. (2019)]. Children develop different abilities at different ages, therefore the impact of specific cognitive processes on sports performance may vary across development, as well as across different sports (Vitali et al., 2019a). Also, research examining sports performance that considered emotions has only rarely included the role of cognitive processes (e.g., Raab et al., 2016). Lastly, research studying individual differences in sports performance only rarely involved youth athletes of team sports, given the difficulty in obtaining valid and ecological individual performance measures, and systematically differentiated between open- and closed-skills sports.

Open-skills sports occur in unpredictable environments, like those involving a direct opponent, either individually (e.g., combat sports, tennis) or in team (e.g., volleyball, rugby). In these sports, the movement cannot be completely programmed in advance and the athlete must constantly adapt to the environment, placing a great load on cognitive functioning (Claver et al., 2016; Gu et al., 2019). In contrast, in closed-skills sports (e.g., artistic gymnastics, shooting) the athlete’s performance is executed as planned and within a stable environment (Voss et al., 2010; Wang et al., 2013). We argue that the role of cognitive and emotional processes in predicting sports performance may critically depend on the environmental (un)stability and the resulting cognitive load [i.e., the amount of working memory resources involved in a task; see Arsalidou et al. (2013)] that characterize these different types of sports.

As anticipated above, a branch of research in sport psychology examines the effects of sport on cognition, focusing on athletes practicing different sports. In general, these studies show the superiority of athletes from interceptive sports (i.e., sports that require coordination between the athletes’ body and an object in the environment) in several cognitive paradigms, both under a sport-specific context [for a meta-analysis, see Mann et al. (2007)] and on general laboratory-based tasks (Voss et al., 2010; Wang et al., 2013). Although the impact of sport in enhancing cognitive abilities is acknowledged, the role of cognition in sports performance is still relatively under-researched, and mainly studied with adult athletes [see Kalén et al. (2021) for a recent meta-analysis].

Developmental studies investigating cognitive functioning in youth athletes are scant and highlight the importance of understanding cognitive functioning in an early phase of development to identify predictors of sports performance. A study testing 8–16-year-old elite soccer players and age-matched non-elite soccer players revealed that measures of inhibition, alerting, and orientation of attention differentiated elite from non-elite players with high accuracy (Verburgh et al., 2014a). Similar results were found in other studies (Huijgen et al., 2015; Vestberg et al., 2017). Specifically, Huijgen et al. (2015) found that elite youth soccer players had better inhibitory control, shifting, and metacognition than their sub-elite counterparts, while Vestberg et al. (2017) found that youth soccer players’ performance at the Design Fluency test (a complex executive task) predicted the number of goals scored during the season. Comparable results were found by Sakamoto et al. (2018) in a study with 8–16-year-old soccer players and by Ishihara et al. (2019) in a longitudinal study with junior league tennis players.

An important variable examined by researchers is working memory (WM). The Theory of Constructive Operators (TCO) (Pascual-Leone and Goodman, 1979; Pascual-Leone, 1987) conceptualizes WM, defined within TCO as M capacity, as executive attention with a limited capacity, and provides a precise developmental model of capacity growth. M capacity is the maximum number of schemes (units of cognition) that one individual can co-activate and increases by one unit every 2 years, starting from 3 years of age. Since performing complex motor skills generates a high cognitive load (due to multiple motor schemes to be co-activated), some authors have studied the role of WM in sport [for a review, see Furley and Memmert (2010)]. Bisagno and Morra (2018) conducted a study on a sample of youth female volleyball players within the TCO framework where WM was the main predictor underlying the correct execution of a series of attacks. Findings showed developmental (age-related) thresholds in performing attack gestures of increasing complexity. Furley and Memmert (2012) found that basketball and hockey adult players with higher WM were better than those with lower WM in focusing attention and producing creative solutions in a decision-making task.

Executive functions (EFs) are further relevant cognitive variables, which can be broadly defined as top-down processes that regulate goal-directed, controlled behaviors [Espy, 2004; see also Diamond (2013)]. A prominent theoretical account of EFs was proposed by Miyake et al. (2000) who identified three distinct but interrelated EFs, namely (i) inhibition, which concerns both the ability to suppress a preponderant or automatic response and the ability to suppress interfering mental representations and distracting stimuli; (ii) updating, that refers to the active updating of the information in WM; and (iii) shifting, which is described as the ability to flexibly move from one mental set to another. Research on the role of EFs in sports performance has mainly focused on inhibition. In a study with youth soccer players (Verburgh et al., 2014a), high talented athletes performed significantly better than amateurs of the same age in inhibitory tasks. Similar results were observed with volleyball players (Lipoma et al., 2006), and in other open-skills sports (Wang et al., 2013). Literature related to sports performance concerning shifting and updating is scarce. Krenn et al. (2018) compared volleyball players with athletes of less strategic sports on an N-back task (updating), finding a better response time for the first. Indirectly suggesting a role of shifting, some studies highlighted an advantage of volleyball players in re-orienting attention (e.g., Castiello and Umiltà, 1990). There is a need to consider jointly the three EFs to understand their role in sports performance.

Another relevant variable is attentional style, defined as the individual’s disposition to preferably adopt a certain attentional focus (Nideffer, 1976). The theory of attentional style and the test derived from it [Test of Attentional and Interpersonal Style, TAIS (Nideffer, 1976, 2002)], largely used in sport psychology, were developed to provide a framework for understanding and predicting the conditions under which individuals perform at their best. According to Nideffer, attention has two bipolar, orthogonal dimensions: width and direction. Crossing them, four attentional styles are identified: (i) focused or narrow external; (ii) aware or broad external; (iii) systematic or narrow internal; and (iv) strategic or broad internal. Nideffer (1990) suggested that, according to the type of sport, a style may be preferred to others. In open-skills sports, a “broad and external” focus would be more useful to respond effectively to sudden stimuli (Bosel, 1998). Conversely, the athletes practicing closed-skills sports would prefer a “narrow and internal” focus (Nideffer, 2007). The relation between attentional styles and sports performance has been widely debated in the literature (Vallerand, 1983; Kerr and Cox, 1990). Therefore, we tested the attentional style as a predictor of sports performance.

Taken alone, however, the general cognitive prerequisites described above (i.e., WM capacity, EFs, and attentional style) are not sufficient to explain sports performance, which is also highly impacted by emotional aspects. Emotions are complex and organized response patterns, favoring one individual’s adaptation to the environment (Ekman, 1992). The Circumplex model of emotions (Russell, 1980) proposes that affective states arise from the appraisal of sensations based on arousal (high vs. low) and hedonic tone (pleasant vs. unpleasant). Athletes are usually able to easily express how they feel in terms of pleasant or unpleasant emotions even under pressure (Vitali et al., 2019b).

Emotions in sport have been studied over the past 50 years, highlighting the importance of both pleasant and unpleasant emotions on specific components of performance (e.g., attention and memory). Research showed how some emotions typically considered dysfunctional (e.g., anxiety and anger) can be predictive of a good performance for certain athletes and types of sports, even if unpleasant (Campo et al., 2012; Ruiz et al., 2016). On the other hand, Mahoney and Avener (1977) observed that disciplines requiring high precision, like gymnastics, are facilitated by low-arousal emotions. These studies suggest that the role of emotions related to sports performance is not invariant but rather linked to the type of sport. Athletes of open-skills sports may benefit more from high-arousal emotions, even if unpleasant (e.g., anxiety), to better react to rapid environmental changes. Conversely, athletes of closed-skills sports could profit from low-arousal emotions since they need to calmly focus on their bodily control.

An important drawback of existing research is that the additive and/or interactive role of cognition and emotions has rarely been studied together. However, some studies suggest that emotions with high arousal and unpleasant hedonic tone (e.g., anxiety) are debilitating (Hill et al., 2010), and can lead the athlete to choke under pressure since they burden WM by generating intrusive thoughts (Baumeister, 1984), or because athletes use part of their WM to inhibit these feelings (Beilock, 2007). We argue that this examination is key to future research because both cognitive and affective factors influence sports performance and examining them in isolation can result in biased estimates of their reciprocal role.

We, therefore, conducted a cross-sectional study with a group of youth female athletes to test cognitive and emotional processes as predictors of sports performance. We examined the additive role of these factors as well as the interplay between WM and emotions. Crucially, we considered two different types of sport, that is, an open-skills sport (i.e., volleyball) and a closed-skills sport (i.e., artistic gymnastics). The purpose of this study was three-fold: (i) applying a developmental framework to the study of the predictors of sports performance; (ii) investigating both cognition and emotions in relation to sports performance; and (iii) comparing open- and closed-skills sports. In particular, we considered WM within the TCO framework, EFs, and attentional style whose addictive effect has never been studied to the best of our knowledge. We also focused on arousal and hedonic tone of pleasant and unpleasant emotions according to the Circumplex model. For both volleyball players and artistic gymnasts, we based the performance measures on the participants’ actual competitions during the 2017–2018 sports year.

The hypotheses of our study were the following:

(H1) M capacity was expected to predict performance for volleyball players but not for artistic gymnasts because volleyball players are expected to deal with a greater cognitive load during performance (e.g., Claver et al., 2016).

(H2) Inhibition was expected to predict volleyball players’, but not artistic gymnasts’ performance (e.g., Wang et al., 2013). Predictiveness of updating and shifting was studied in an exploratory way.

(H3) A broad-external attentional focus was expected to predict volleyball players’ performance, while a narrow-internal focus was expected to be predictive of the artistic gymnasts’ performance (Nideffer, 1990).

(H4) Artistic gymnasts’ performance was expected to be predicted positively and negatively by low arousal-pleasant hedonic tone emotions and high arousal-unpleasant hedonic tone emotions, respectively (e.g., Mahoney and Avener, 1977). High-arousal emotions, both pleasant and unpleasant, were expected to have a positive effect on volleyball players’ performance (e.g., Campo et al., 2012).

(H5) Finally, based on the choking-under-pressure theories (e.g., Hill et al., 2010), we predicted a moderation effect of high arousal-unpleasant hedonic tone emotions on the relation between WM and sports performance.

To the best of our knowledge, no studies have so far investigated performance in youth sport by considering different sports and simultaneously using different cognitive and emotional predictors. Identifying how cognitive and emotional processes may differentially impact sports performance at different ages and for different types of sports can help design both developmentally oriented and sport-specific psychological interventions.

## Materials and methods

### Participants

The sample consisted of 218 youth female athletes, of which 114 volleyball players (Under 12 to Under 18), and 104 artistic gymnasts (23 in the *Gold* and 81 in the *Silver* category), aged between 11 and 17 years (*M* = 14.06 years, *SD* = 1.73), recruited from clubs in Northern Italy. Both sports are predominantly practiced by female athletes. Thus, we included only female participants to avoid gender-related biases. We chose the age range according to two research needs: firstly, guaranteeing a high developmental variability to detect a developmental trajectory in cognitive functioning, and secondly including sufficiently meta-conscious participants for the compilation of self-report measures. Moreover, to ensure that all participants experienced a fair amount of deliberate practice and competitions, we enrolled athletes with at least 3 years of experience in their sport. The two sub-samples of volleyball players and artistic gymnasts were comparable with respect to the age (*t* = 1.72, *p* = 0.09), but not to the years of sport experience (*t* = 5.42, *p* < 0.001), with artistic gymnasts (*M* = 7.56, *SD* = 2.77) having more sports experience than volleyball players (*M* = 5.66, *SD* = 2.36). This is not surprising and coherent with artistic gymnastics being a sport with particularly early specialization.

### Procedure

Data related to cognitive variables were collected from November 2017 to April 2018 by the first author of this article with the help of two trainees during three individual meetings lasting about 40 min with each athlete. The meetings were held in meeting rooms and/or empty locker rooms to guarantee a quiet and appropriate setting for the participants to concentrate. One meeting per week (out of three average training days) was scheduled. A questionnaire regarding emotions was collectively administered within volleyball players’ and gymnasts’ groups, usually in the gym before practice, taking approximately 15 min. All the procedures were conducted according to the Declaration of Helsinki. The study was approved by the ethics committee of the University of Verona (Italy) (DEDIPAC WP3.2). Parents provided informed consent for participation.

### Measures

#### Cognition and emotions assessment

##### working memory

We administered three WM capacity tasks designed consistently with an attention-based view of WM, namely (a) the Mr. Cucumber Test (Case, 1987); (b) the Figural Intersection Test (FIT) (Pascual-Leone and Baillargeon, 1994), which both involve visual material; and (c) the Direction Following Task (DFT) (Pascual-Leone and Johnson, 2005), which involves verbal stimuli of increasing complexity, in its Italian adaptation (Morra et al., 2013). The same measures were used in previous research with youth volleyball players; for details on scoring see Bisagno and Morra (2018).

##### Inhibition

We used two computerized tasks, namely a Color-word Stroop Task (Friedman et al., 2008) implemented in E-Prime, and an Arrow Flanker Task (Ridderinkhof et al., 1997), acquired from Inquisit 5 Lab. In the Stroop task participants are asked to verbally name the color of each stimulus of a series as quickly and accurately as possible. In the incongruent trials, stimuli are color-word printed in a different color, so that the participant needs to inhibit the reading automatism. In the Flaker task, the participant must indicate, by pressing on the keyboard as quickly and accurately as possible, the direction of an arrow flanked by two distractors.

##### Shifting

We adopted a computerized Color/Shape Task (Miyake et al., 2004) administered *via* Inquisit 5 Lab, adapting it to reduce the number of conditions. In each trial of this task, the participants see a shape (i.e., circle or triangle) superimposed on a square (i.e., red or green), and are asked to categorize the stimulus according to the cue presented to them (i.e., the word “shape” or “color”, which appears before the stimulus itself). We also administered a paper-pencil Trail Making Test, parts A and B (TMT) (Reitan, 1958). Within the TMT-A, participants are asked to connect in ascending order 25 digits scattered on the sheet, while in the TMT-B the targets are both numbers and letters, and the participant must alternate them (i.e., 1, A, 2, B, etc.), being as fast and accurate as possible. For both TMT-A and B completion time is recorded by the experimenter. Shifting is calculated as TMT-B completion time minus TMT-A completion time.

##### Updating

We used two computerized tasks, namely a Keep-Track Task (Friedman et al., 2008) implemented in E-Prime and an N-Back Task acquired from Inquisit 5 Lab [adapted from Jaeggi et al. (2010)]. In the Keep Track, fifteen words belonging to six categories are presented serially in random order. Target categories remain visible at the bottom of the screen. The participant’s task is to remember the last word presented per each of the target categories. In the N-back, participants are shown a sequence of shapes and are asked to press on the keyboard each time the current stimulus is identical to the one presented in *N* positions earlier. There are three levels of increasing difficulty, from 2 to 4-back. In our task, the stimuli were simplified compared to the original version to be better suitable for our younger participants [Jaeggi et al. (2010) tested young adults].

##### Attentional style

We administered the short Italian version of the TAIS (Lipoma et al., 2006) twice (T1 and, after 1 month, T2). The test consists of twelve items (measured on a 0–4 Likert scale), and it is composed of six subscales of two items each, namely Broad External Focus (BET, α = 0.79; e.g., “In a room full of children or on a playground, I can keep track of what everyone is doing.”), Broad Internal Focus (BIT, α = 0.79; e.g., “I can generate many ideas with just a little information.”), Overload of External or Internal stimuli (OET, α = 0.69; “When people talk to me, I am distracted by what I hear and the things around me.” and OIT, α = 0.82; “When people talk to me, I am distracted by my thoughts and ideas.”), Narrow focus (NAR, α = 0.64; “I find it easy to prevent my thoughts from interfering with what I am listening or watching.”), and Reduced focus (RED, α = 0.76; “I find it difficult to clear my mind of a thought or idea.”). The overall score on each scale is the sum of the scores on two items.

##### Emotions

We created an *ad-hoc* short 12-items “Circumplex” questionnaire. To do so, based on Russell’s (1980) conceptualization, we selected the 12 most used emotion descriptors, three from each quadrant deriving from the interaction between arousal and hedonic tone dimensions. The descriptors were presented to participants as a list and the players were asked to evaluate (on a 1 to 7 Likert scale) how often, in their general experience as athletes, before a competition they felt: tense, stressed, angry (high-arousal, unpleasant hedonic tone; α = 0.65); discouraged, depressed, tired (low-arousal, unpleasant hedonic tone; α = 0.55); serene, relaxed, calm (low-arousal, pleasant hedonic tone; α = 0.81); and stimulated, excited, happy (high-arousal, pleasant hedonic tone; α = 0.72). The confirmatory factor analyses that detail how we derived the emotional predictors are presented in the Results Section “Emotion-related measures.”

#### Performance measures

For what concerns volleyball players, we video-recorded at least three matches for each player during the 2017–2018 championship. Except for isolated cases (e.g., equipment malfunctioning), the matches recorded for each team were all those of the “second round” of the sporting season. Two raters (both volleyball coaches) independently evaluated each athlete’s performance for every single contact with the ball, according to a-priori-defined criteria. Specifically, every time that a participant made contact with the ball (i.e., serve, pass, set, or hit—the block was not scored unless it ended the action), that skill was evaluated with the attribution of 0 points, 0.5 points, or 1 point that were in the end summed up for each set. From these scores, an individual performance ratio index (IR) was calculated for each participant. We also collected team performance (TP) for each Set. The final weighted index of performance (WIP) was calculated for both observers by saving the residuals of the regression of IR on TP. Full details about this procedure can be found in Bisagno et al. (2019).

Regarding artistic gymnasts, we collected for each participant all scores on all the apparatus (i.e., balance beam, floor, uneven bars, and vault) in individual and team competitions held in 2017–2018.

### Data analyses

As preliminary analyses, Exploratory (EFA) and Confirmatory Factor Analyses (CFA) using SPSS 25 (IBM, Armonk, NY, United States) and LISREL 8.80 were conducted to identify latent variables from the different observed variables from cognitive, attentional, and emotion-related measures. Regarding performance measures, an individual index of performance for each volleyball player (Bisagno et al., 2019), as well as for each gymnast was calculated.

To test the hypotheses coherently with a developmental framework, a series of hierarchical regression analyses with Performance measures as dependent variables were used, adopting a stepwise method. To test the “choking under pressure” hypothesis, moderation analysis was conducted. Specifically, regression was applied using the PROCESS version 3.5 computational tool for SPSS by Hayes (2022, Model 1); bootstrapping procedures with 5,000 resamples were used to test the significance of the indirect effects. Additional analyses were performed to explore different possible links between emotions and cognition by looking specifically at the role of arousal. To do so, we calculated two indices (i.e., scores on High+ *minus* scores on Low+) and arousal in unpleasant emotions (i.e., scores on High− *minus* scores on Low−). We then divided the volleyball athletes into four groups, according to whether their scores were above or below the mean score in both indices, as follows:

• Group A: below the mean in arousal in both + and − emotions;
• Group B: below the mean in arousal in + but above the mean in − emotions;
• Group C: below the mean in arousal in − but above the mean in + emotions;
• Group D: above the mean in arousal in both + and − emotions.

We then calculated the correlations between the Mcap-Upd and the volleyball performance within each group.

As additional analyses, we explored the differences between age groups and sports, as well as their interaction. A 3 × 2 (11–12/13–14/15–17 years old × volleyball/artistic gymnastics) multivariate analysis of variance (MANOVA) was conducted on all predictors. Follow-up analysis of variance (ANOVA) was then used to identify significant differences.

### Results

#### Cognitive measures

Based on the EFA and CFA, with respect to the cognitive variables, the best fitting model was a two-factor model assuming a M capacity-Updating factor and an Inhibition-Shifting one [χ^2^(53) = 63.73, *p* = 0.13, RMSEA = 0.04, SRMR = 0.05, CFI = 0.98, GFI = 0.95]. Loadings are presented in Table 1. From the factor scores of the observed variables, we then derived two composite predictors, namely an M capacity-Updating (Mcap-Upd) factor, and an Inhibition-Shifting factor (Inh-Shift).

**TABLE 1:** Lambda-X matrix of the factor loadings for the two-factor model of general cognitive measures.

	M Cap-Upd	Inh-Shift
Cucumber	0.50	—
	(0.07)	
	7.09	
DFT	0.69	—
	(0.07)	
	10.55	
FIT	0.68	—
	(0.07)	
	10.35	
Keep-Track errs.	−0.52	—
	(0.07)	
	−7.41	
N-Back errs.	−0.63	—
	(0.07)	
	−9.43	
Stroop errs.	—	0.43
		(0.07)
		5.81
Stroop cost	—	0.17
		(0.07)
		2.20
Flanker errs.	—	0.42
		(0.07)
		5.59
Flanker cost	—	0.24
		(0.08)
		3.17
C/S errs.	—	0.73
		(0.07)
		10.10
C/S cost	—	0.05
		(0.08)
		0.68
TMT cost	—	0.42
		(0.07)
		5.30
Φ (PHI) = −0.82 (0.06); −13.48		

For each parameter, the Table shows the estimated value (the standard error), and the corresponding z. The C/S cost had a non-significant load on the Ihn-Shift factor. For this reason, we also computed a CFA with only eleven variables. The results did not differ, and the C/S cost was, therefore, not excluded.

For the TAIS measures, the best fitting model comprised four factors [χ^2^(45) = 55.76, *p* = 0.13, RMSEA = 0.03, SRMR = 0.04, CFI = 0.99, GFI = 0.96]: External Attentional Style Focus (Ext. Focus); Internal Attentional Style Focus (Int. Focus); Narrow Attentional Style Focus (Narrow Focus), and a Dysfunctional Attentional Style (Dysfunctional), given by the mean score of all the dysfunctional attentional focuses (OIT, OET, RED subscales) at both T1 and T2 (see Table 2 for the loadings).

**TABLE 2:** Lambda-X matrix of the factor loadings for the four-factor model of the attentional style predictors.

	External focus	Internal focus	Narrow focus	Dysfunctional style
BET T1	0.72	—	—	—
	(0.08)			
	9.14			
OET T1	—	—	—	0.56
				(0.07)
				7.63
BIT T1	—	0.89	—	—
		(0.09)		
		9.56		
OIT T1	—	—	—	0.67
				(0.07)
				9.32
NAR T1	—	—	0.78	
			(0.11)	
			6.59	
RED T1	—	—	—	0.60
				(0.07)
				8.20
BET T2	0.92	—	—	—
	(0.08)			
	9.14			
OET T2	—	—	—	0.64
				(0.07)
				8.88
BIT T2	—	0.73	—	—
		(0.09)		
		8.53		
OIT T2	—	—	—	0.65
				(0.07)
				9.03
NAR T2	—	—	0.60	—
			(0.10)	
			6.23	
RED T2	—	—	—	0.65
				(0.07)
				9.11

For each parameter, the Table shows the estimated value (the standard error), and the corresponding z.

#### Emotion-related measures

Confirmatory factor analyses for the emotion-related measures indicated as the best-fitting a four-factor model, coherent with Russel’s model [χ^2^(48) = 136.72, *p* < 0.001, RMSEA = 0.09, SRMR = 0.08, CFI = 0.93, GFI = 0.91]. The first factor included low arousal and pleasant hedonic tone emotions, with the highest loading for “Relaxed” (λ = 0.84). The second factor included low arousal and unpleasant hedonic tone emotions, with the highest loading for “Discouraged” (λ = 0.63). The third factor included high arousal and pleasant hedonic tone emotions, with the highest loading for “Happy” (λ = 0.78). Lastly, the fourth factor included high arousal and unpleasant hedonic tone emotions, with the highest loading for “Stressed” (λ = 0.80) (see Table 3 for all loadings). We derived four predictors from the “Circumplex” questionnaire: mean score of the emotions with low arousal and pleasant hedonic tone (Low+: Serene, Relaxed, Calm); mean of the emotions with low arousal and unpleasant hedonic tone (Low−: Discouraged, Depressed, Tired); mean of the emotions with high arousal and pleasant hedonic tone (High+: Stimulated, Excited, Happy); and mean of the emotions with high arousal and unpleasant hedonic tone (High−: Tense, Stressed, Angry).

**TABLE 3:** Lambda-X matrix of the factor loadings for the four-factor model of the emotions predictors.

	High arousal	High arousal	Low arousal	Low arousal
	Hedonic tone −	Hedonic tone +	Hedonic tone −	Hedonic tone +
Stressed	0.80	—	—	—
	(0.06)			
	13.04			
Relaxed	—	—	—	0.84
				(0.06)
				14.64
Downhearted	—	—	0.63	—
			(0.08)	
			7.65	
Happy	—	0.78	—	—
		(0.08)		
		9.92		
Tense	0.73	—-	—	—
	(0.06)			
	11.73			
Tired	—	—	0.39	—
			(0.08)	
			4.96	
Excited	—	0.44	—	—
		(0.08)		
		5.85		
Calm	—	—	—	0.82
				(0.06)
				14.14
Stimulated	—	0.59	—	—
		(0.08)		
		7.85		
Depressed	—	—	0.45	.—
			(0.08)	
			5.66	
Angry	0.20	—-	—	—
	(0.07)			
	2.78			
Serene	—	—	—	0.66
				(0.06)
				10.58

As control variables, we included age and experience in years of sport practice. Descriptive characteristics of final predictors are presented in Table 4.

**TABLE 4:** Observed variables and derived predictors descriptive characteristics.

Raw data	Mean	St. dev.	Derived predictor	Mean	St. dev.
Mr Cucumber	6.01	1.18	Mcap-Upd	0.00	0.71
DFT	5.26	1.30			
FIT	6.09	1.34			
Keep track (errors %)	0.35	0.12			
N-back (errors %)	0.27	0.08			
Stroop (incongruent errors)	3.07	2.64	Inh-Shift	0.00	0.60
Stroop cost (milliseconds)	143.30	70.00			
Flanker (incongruent accuracy)	0.97	0.04			
Flanker cost (milliseconds)	55.33	36.91			
Color/Shape (shift errors)	0.29	0.12			
Color/Shape cost (milliseconds)	123.32	114.06			
TMT cost (seconds)	30.24	18.93			
BET	4.84	1.36	Ext. focus		
BIT	4.94	1.41	Int. focus		
NAR	3.93	1.68	Narrow focus		
OIT	3.53	1.73	Dysfunctional	3.43	1.16
OET	3.22	1.44			
RED	3.51	1.57			
Stressed	4.33	1.73	High−	4.48	1.36
Tense	5.27	1.64			
Angry	2.27	1.42			
Happy	5.12	1.44	High+	4.93	1.11
Excited	4.55	1.54			
Stimulated	4.91	1.41			
Depressed	1.55	0.90	Low−	2.34	0.87
Discouraged	2.66	1.31			
Tired	2.76	1.44			
Relaxed	2.90	1.41	Low+	3.16	1.36
Calm	2.81	1.66			
Serene	3.92	1.71			

“Mcap-Upd” and “Inh-Shift” have mean zero because they are factorial scores.

#### Performance measures

For each volleyball player, we calculated a weighted individual index of performance (WIIP). For both observers, the WIIP index was given by the residuals of the regression of the participant’s individual ratio (IR) points on ball touches on the weighted game (WG) index. This procedure was meant to weigh the individual volleyball player’s performance on team performance. We then assessed the inter-rater agreement between the two judges, namely *r* = 0.84, *p* < 0.001. Therefore, for volleyball performance, we computed a final performance measure for each volleyball player using the mean WIIP between Observer 1 and Observer 2 (Volleyball Performance). A detailed description of the methodology used in the present study is presented in Bisagno et al. (2019).

With respect to the artistic gymnasts’ performance, we computed z-scores for each apparatus (i.e., balance beam, floor, uneven bars, and vault). The final performance measure for each artistic gymnast is the mean of her z-scores (Gymnastics Performance).

### Main results

#### Volleyball players

As can be seen in Table 5, in the volleyball players sample, even controlling for age and sport experience, the correlation between the Mcap-Upd factor and the Inh-Shift remained moderately negative (*r* = −0.58, *p* < 0.001). More importantly, the Mcap-Upd factor was the only variable with a significant (weak to moderate) correlation with the volleyball players’ performance (*r* = 0.33, *p* < 0.001). This is preliminary evidence for the involvement of general cognitive resources in sport performance.

**TABLE 5:** Zero-order and partial correlations between predictors and the volleyball players’ performance.

	[1] Mcap-Upd	[2] Inh-Shift	[3] Ext. focus	[4] Int. focus	[5] Narrow focus	[6] Dysfunc. focus	[7] High+ emotions	[8] High – emotions	[9] Low+ emotions	[10] Low− emotions	[11] Volley perf.	Age	Years exp.
[1]	1	−0.607***	0.213*	0.222*	0.202*	–0.167	0.034	0.018	0.037	0.039	0.323***	0.385***	0.205*
[2]	−0.575***	1	–0.079	–0.046	−0.210*	–0.010	0.206*	–0.108	–0.008	–0.157	–0.179	−0.256***	−0.188*
[3]	0.243*	–0.081	1	0.247**	0.098	−0.197*	0.282**	–0.106	0.131	−0.242**	0.177	0.001	0.062
[4]	0.267**	–0.056	0.242*	1	0.238*	–0.127	0.108	–0.094	0.118	–0.086	–0.027	–0.035	0.028
[5]	0.237*	−0.227*	0.098	0.237*	1	−0.354***	0.129	–0.184	0.217*	−0.264**	–0.032	–0.036	–0.018
[6]	−0.223*	0.022	−0.210*	–0.133	−0.356***	1	–0.161	0.295**	−0.308**	0.352***	–0.014	0.120	0.156
[7]	0.002	0.238*	0.283**	0.110	0.133	–0.176	1	−0.217*	0.299**	−0.466***	–0.042	0.087	0.072
[8]	–0.004	–0.097	–0.105	–0.091	–0.182	0.294**	−0.223*	1	−0.729***	0.487***	0.117	0.054	0.030
[9]	0.041	–0.002	0.125	0.114	0.218*	−0.324***	0.298**	−0.732***	1	−0.310**	–0.002	0.021	0.071
[10]	–0.021	–0.125	−0.244**	–0.081	−0.261**	0.344***	−0.485***	0.485***	−0.316**	1	–0.009	0.145	0.091
[11]	0.330***	–0.157	0.166	–0.037	–0.031	–0.047	–0.056	0.115	–0.019	–0.024	1	0.116	0.203*

Zero-order (Pearson) correlations above diagonal. Partial correlations controlled for age and years of sports experience below diagonal. *p < 0.05, **p < 0.01, ***p < 0.001.

We, therefore, ran a hierarchical regression analysis with Volleyball Performance as the dependent variable. Based on a developmental rationale, we entered age and years of sport experience as a first block, Mcap-Upd, and Inh-Shift, which are cognitive functions with a developmental trajectory in a second block, and the attentional styles and emotion-related variables in a third block using a stepwise method. This model accounted for 15% of the variance. Coherently with our predictions, the Mcap-Upd factor was the main predictor (ß = 0.38, *p* < 0.001). The years of sport experience had a ß of 0.28, *p* = 0.020, while age was non-significant. Subsequently, we also ran a regression analysis entering all the twelve variables (Table 6) in one block, which explained 23% of the variance. Again, the main predictor was Mcap-Upd (ß = 0.45, *p* = 0.001), together with the years of sport experience (ß = 0.29, *p* = 0.030), and high arousal and unpleasant hedonic tone emotions (ß = 0.30, *p* = 0.029). This predictor became significant with all predictors entered simultaneously and, therefore, controlling for each other and this result was consistent with the idea that high-arousal emotions were predictive of better outcomes in open-skills sports. These results confirmed H1 and H4. Contrary to H2 and H3, Inh-Shift was not included among the predictors, as well as the attentional styles. Taken these analyses together, the prominent role of WM and updating as predictors of performance in youth volleyball players was the main finding regarding this subsample.

**TABLE 6:** Regression analysis with the volleyball performance as the dependent variable, and all predictors and control variables entered in the equation.

Predictors		Volleyball performance R^2^ = 0.23
		B
General cognitive abilities	M capacity-updating	0.446**
	Inhibition-shifting	0.115
Attentional style	External	0.131
	Internal	–0.138
	Narrow	–0.084
	Dysfunctional	0.032
Emotions	High+	–0.167
	High−	0.302*
	Low+	0.209
	Low−	–0.175
Control variables	Age	–0.228
	Years of experience	0.291*

*p < 0.05, **p < 0.01.

Based on H5, we then tested possible moderation effects of emotions on the relation between WM and performance. First, we tested the “choking under pressure” hypothesis. We considered Volleyball Performance as the dependent variable, Mcap-Upd as the independent variable, and emotions with high arousal and unpleasant hedonic tone (High−) as the moderator. Neither the High− factor [B = 0.01 (0.01), *p* = ns, 95% CI [−0.005, 0.019]], nor the interaction [B = 0.00 (0.01), *p* = ns, 95% CI [−0.020, 0.013]] accounted for further variance. Thus, we found no evidence of a detrimental effect of the High− emotions on the relationship between WM and performance and, therefore, H5 was not supported. Moving from these unexpected results, we then further explored possible links between emotions and cognition with additional analyses by looking at the effect of arousal in the four groups described in the Data Analyses section:

• Group A: *n* = 40;
• Group B: *n* = 20;
• Group C: *n* = 12;
• Group D: *n* = 42.

In groups A and B, the Mcap-Upd and the volleyball performance were uncorrelated (*r* = 0.15 and *r* = 0.26, respectively). In group C, the correlation was moderately high (*r* = 0.64, *p* = 0.020), while in group D it was low (*r* = 0.32, *p* = 0.039). These findings suggested that emotional arousal played a role in moderating the relationship between WM and volleyball performance not in isolation, but in a specific combination with the hedonic tone. In other words, we did not find a direct moderating effect of High− emotions on the relationship between WM and performance but there was evidence of a moderating effect of specific joint emotional patterns.

#### Artistic gymnasts

We looked at the correlations between cognitive and emotional measures as predictors of artistic gymnasts’ performance (Table 7). The emotional predictors were the only variables related to the artistic gymnasts’ performance: Low+ emotions (i.e., serene, relaxed, calm) showed a positive correlation (*r* = 0.25, *p* = 0.011), while High− emotions (i.e., tense, stressed, angry) showed a negative correlation (*r* = −0.33, *p* < 0.001) with the gymnast’ scores.

**TABLE 7:** Zero-order and partial correlations between predictors and the artistic gymnasts’ performance.

	[1] Mcap-Upd	[2] Inh-shift	[3] Ext. focus	[4] Int. focus	[5] Narrow focus	[6] Dysfunc. focus	[7] High+ emotions	[8] High – emotions	[9] Low+ emotions	[10] Low− emotions	[11] Gym perf.	Age	Years exp.
[1]	1	−0.554***	0.242*	0.077	0.007	–0.072	0.017	0.142	–0.082	–0.044	0.056	0.463***	0.352***
[2]	−0.436***	1	−0.237*	–0.110	–0.114	–0.013	–0.027	–0.161	0.032	0.060	–0.038	−0.403***	−0.410***
[3]	0.202*	–0.193	1	0.378***	0.352***	–0.338	0.192	–0.029	0.150	–0.181	0.018	0.123	0.135
[4]	0.130	–0.143	0.391***	1	0.156	−0.247*	0.258**	0.012	0.002	–0.078	–0.061	–0.109	0.047
[5]	0.047	–0.158	0.365***	0.145	1	–0.184	–0.194	–0.054	0.038	–0.087	–0.024	–0.084	–0.014
[6]	–0.182	0.072	−0.371***	−0.235*	–0.172	1	–0.075	0.189	–0.062	0.276**	–0.017	0.182	0.104
[7]	0.028	–0.033	0.194	0.255*	−0.198*	–0.072	1	–0.090	0.257**	−0.235*	–0.035	–0.023	0.015
[8]	–0.026	–0.010	–0.082	0.043	–0.030	0.138	–0.090	1	−0.556***	0.369***	−0.342***	0.335**	0.243*
[9]	–0.008	–0.031	0.172	–0.024	0.021	–0.032	0.255*	−0.543***	1	−0.219*	0.275**	–0.175	–0.052
[10]	–0.122	0.140	−0.205*	–0.075	–0.081	0.261**	−0.237*	0.349***	−0.208*	1	–0.124	0.117	0.120
[11]	0.139	–0.071	0.023	–0.121	–0.052	0.018	–0.050	−0.331***	0.245*	–0.125	1	–0.192	0.101

Zero-order (Pearson) correlations above diagonal. Partial correlations controlled for age and years of sports experience below diagonal. *p < 0.05, **p < 0.01, ***p < 0.001.

Similarly to the volleyball players, we ran a regression analysis entering age and years of sport experience as a first block, Mcap-Upd and Inh-Shift in a second block, and the attentional styles, and emotion-related variables in a third block with a stepwise method. The model (R^2^ = 0.20) showed that the high-arousal unpleasant emotions were the main predictor (ß = −0.34, *p* = 0.001), followed by the years of sports experience (ß = 0.32, *p* = 0.003) and by age, which were negative predictors (ß = −0.25, *p* = 0.023). This could be due to the high portion of variance shared with the years of sports experience and means that younger artistic gymnasts either performed generally better than older ones or were judged more favorably. In a second regression analysis (Table 8) we entered all the twelve variables at once. This model accounted for 25% of the variance, and the main predictor was, in this case, age (ß = −0.36, *p* = 0.005), followed by years of sport experience (ß = 0.30, *p* = 0.011) and high arousal and unpleasant hedonic tone emotions (ß = −0.27, *p* = 0.030).

**TABLE 8:** Regression analysis with the artistic gymnastics performance as the dependent variable, and all predictors and control variables entered in the equation.

Predictors		Gym performance R^2^ = 0.25
		**B**

General cognitive abilities	M capacity-updating	0.145
	Inhibition-shifting	–0.035
Attentional style	External	0.060
	Internal	–0.092
	Narrow	–0.093
	Dysfunctional	0.067
Emotions	High+	–0.113
	High−	−0.270*
	Low+	0.110
	Low−	–0.034
Control variables	Age	−0.360**
	Years of experience	0.296*

*p < 0.05, **p < 0.01.

In line with H1, Mcap-Upd did not predict the performance for artistic gymnasts (while it did for volleyball players). Interestingly and partly in accordance with H4, High− emotions (but not Low+ emotions) were the only significant psychological predictors of gymnasts’ performance. As further support to our hypothesis that emotions play a key role in determining artistic gymnasts’ performance, we additionally examined if they discriminated between higher and lower-level athletes. We, therefore, ran *t*-tests to detect potential differences between Gold and Silver artistic gymnasts with respect to the predictors. The only significant differences were indeed found in emotions, namely Low+ (*t* = 2.68, *p* = 0.030) and High− (*t* = −3.63, *p* < 0.001). In competition, Silver athletes experienced less Low+ (*M*_*G*_ = 3.17, *M*_*S*_ = 2.40), and more High− (*M*_*G*_ = 4.25, *M*_*S*_ = 5.20). Consistent with the previous regression analyses, this pattern can be interpreted as further proof that emotional control was the main psychological prerequisite for success in artistic gymnastics.

### Additional results

In the end, we analyzed in an exploratory fashion the differences between age groups and sports, as well as their interaction with a 3 × 2 [11–12/13–14/15–17 years old × volleyball/artistic gymnastics] multivariate analysis of variance (MANOVA) on all predictors. Findings revealed significant multivariate effects for 11–12 vs. 13–14 and 15–17 years old, Wilks λ = 0.68, *F*_(20,418)_ = 4.53, *p* < 0.001, ω_*p*_^2^ = 0.14 (large effect size), and for volleyball vs. artistic gymnastics, Wilks λ = 0.78, *F*_(10,209)_ = 5.95, *p* < 0.001, ω_*p*_^2^ = 0.18 (large effect size), but no “sport × age group” interaction, Wilks λ = 0.90, *F*_(20,418)_ = 1.15, *p* = ns, ω_*p*_^2^ = 0.01. Follow-up ANOVAs revealed significant univariate differences between age groups (see Table 9). Coherently with their developmental nature, age-related differences were found with respect to Mcap-Upd and Inh-Shift. Specifically, the 15–17-year-olds showed more efficient working memory and executive functioning than the 11–12- and 13–14-year-old athletes (both *p* < 0.001, medium-to-large effect sizes). Moreover, univariate differences were found on Int. Focus, as well as on High− emotions and Low− emotions. The older age group seemed to adopt an internal attentional focus more than the younger groups (both *p* < 0.050, small effect size). This group also experienced less High− but more Low− emotions prior to competition compared to younger athletes (both *p* < 0.050, small effect size).

**TABLE 9:** Univariate comparisons between 11–12 years old (*n* = 72), 13–14 years old (*n* = 76), and 15–17 years old (*n* = 76) on all the predictors.

	Age group	Mean	Std. dev.	*F*	ω_*p*_^2^
M Cap-Upd	11–12 years old	−0.33	0.59	23.858***	0.171
	13–14 years old	−0.08	0.66		
	15–17 years old	0.39	0.68		
Inh-Shift	11–12 years old	0.17	0.55	11.257***	0.083
	13–14 years old	0.08	0.66		
	15–17 years old	−0.24	0.49		
External	11–12 years old	4.66	1.17	1.294	0.003
	13–14 years old	4.96	1.33		
	15–17 years old	4.90	1.17		
Internal	11–12 years old	4.83	1.36	4.408*	0.029
	13–14 years old	5.26	1.16		
	15–17 years old	4.71	1.31		
Narrow	11–12 years old	4.01	1.52	0.783	−0.002
	13–14 years old	4.02	1.47		
	15–17 years old	3.77	1.33		
Dysfunctional	11–12 years old	3.24	1.18	2.395	0.012
	13–14 years old	3.39	1.18		
	15–17 years old	3.65	1.09		
High+	11–12 years old	4.93	1.15	0.179	−0.007
	13–14 years old	4.87	1.11		
	15–17 years old	4.98	1.08		
High−	11–12 years old	4.43	1.34	5.291**	0.032
	13–14 years old	4.22	1.39		
	15–17 years old	4.80	1.31		
Low+	11–12 years old	3.18	1.28	0.740	−0.002
	13–14 years old	3.24	1.39		
	15–17 years old	3.06	1.41		
Low−	11–12 years old	2.28	0.84	4.565*	0.031
	13–14 years old	2.15	0.87		
	15–17 years old	2.57	0.86		

*p < 0.05, **p < 0.01, ***p < 0.001.

Regarding sports, differences emerged between volleyball players and artistic gymnasts with respect to Inh-Shift, High+ emotions, High− emotions, and Low+ emotions (see Table 10). Specifically, artistic gymnasts showed better inhibitory and shifting skills (*p* < 0.050, small effect size). Volleyball players, on the other hand, reported less High− emotions (*p* < 0.001, medium effect size), suggesting they were less stressed and tense before competing. Volleyball players also reported more pleasant emotions, both with high (*p* < 0.010, low effect size), and low arousal (*p* < 0.001, large effect size).

**TABLE 10:** Univariate comparisons between artistic gymnasts (*n* = 104) and volleyball players (*n* = 114) on all the predictors.

	Sports	Mean	Std. dev.	*F*	ω_*p*_^2^
M Cap-Upd	Gymnastics	−0.03	0.68	0.168	−0.003
	Volleyball	0.03	0.74		
Inh-Shift	Gymnastics	−0.08	0.57	4.448*	0.014
	Volleyball	0.08	0.61		
External	Gymnastics	4.68	1.22	3.230	0.010
	Volleyball	4.98	1.23		
Internal	Gymnastics	4.79	1.19	2.894	0.008
	Volleyball	5.06	1.35		
Narrow	Gymnastics	3.89	1.40	0.187	-0.004
	Volleyball	3.97	1.47		
Dysfunctional	Gymnastics	3.54	1.05	1.977	0.004
	Volleyball	3.33	1.25		
High+	Gymnastics	4.67	1.03	10.336**	0.040
	Volleyball	5.15	1.14		
High−	Gymnastics	4.99	1.17	32.982***	0.121
	Volleyball	4.04	1.37		
Low+	Gymnastics	2.57	1.03	45.056***	0.164
	Volleyball	3.68	1.40		
Low−	Gymnastics	2.40	0.87	1.112	0.000
	Volleyball	2.29	0.88		

*p < 0.05, **p < 0.01, ***p < 0.001.

## Discussion

In this study we aimed to examine the role of cognitive and emotional predictors on sports performance in two sub-samples of youth female athletes practicing volleyball or artistic gymnastics. The Mcap-Upd factor emerged as the main variable that positively predicted successful performance in volleyball. Conversely, High− emotions was the only variable that negatively predicted artistic gymnasts’ performance. This contrast between volleyball players’ performance, for which the main predictor was represented by a general cognitive construct, and artistic gymnasts’ performance, for which the main predictor was represented by their emotional pattern, was consistent with our first hypothesis implying a lower cognitive load for closed-skills sports (Claver et al., 2016).

In volleyball, which is an open-skills sport, the player cannot fully rely on automatisms, because the surrounding environment changes constantly and the athlete is forced to adapt to new stimuli constantly and rapidly. The ability to coordinate and integrate mental schemes becomes fundamental, as well as the ability to update them. This result was also coherent with and added up to other studies that explored the role of WM in motor learning in youth athletes (e.g., Bisagno and Morra, 2018), and decision making in sport (e.g., Furley and Memmert, 2012). Further explanation of the different roles that WM plays in predicting performance in volleyball and artistic gymnastics is offered by dual-process theories (Furley et al., 2015). According to dual-processes theories, Type 1 processes are autonomous and do not rely on WM. In contrast, Type 2 processes require WM to produce an appropriate response to environmental stimuli. What makes a difference between closed- and open-skills sports is also the number of situations in which Type 2 processes are activated. So, if in open-skills sports like volleyball Type 2 processes are heavily involved, in closed-skills sports like artistic gymnastics the performance consists of highly trained routines that do not require a high amount of attentional control. On the contrary, this might even disrupt a fluent execution, causing the so-called “paralysis by analysis” (Baumeister, 1984), which occurs when overinvesting cognitive resources leads athletes to struggle in performing how they otherwise perform routinely.

The Inh-Shift factor predicted neither the artistic gymnasts’ nor, contrary to H2, the volleyball players’ performance. This result was partially in contrast with studies finding a superiority of open-skills sports’ athletes on inhibition (e.g., Wang et al., 2013). Possible explanations for this discrepancy relied on the specific measures we used to test inhibition (previous studies mainly used only response inhibition tasks), or on the factor structure that emerged, which combines inhibition and shifting tasks together. Contrary to H3, also attentional styles were not predictive. This possibly depended on the young age of our sample. Previous studies only investigated elite adult samples (e.g., Nideffer, 1990). Findings suggest that the type of sport can “shape” the attentional focus, so that certain attentional patterns emerged only in elite athletes or specific sports. Still, our research aligned with those studies that do not identify specific attentional patterns as predictors of sports performance in certain sports (e.g., Vallerand, 1983).

In line with H4 and previous research (e.g., Mahoney and Avener, 1977; Pellizzari et al., 2011), the best predictor of artistic gymnasts’ performance was High− emotions, which entered the model with a negative coefficient. This result suggested that what makes the difference in artistic gymnastics was indeed the ability to deal with pre-competitive stress. Another hint in this direction emerged from the comparison between categories, showing that emotional patterns also distinguished between Silver and Gold athletes. The first ones appeared to experience less Low+ and more High− emotions. Since Gold athletes were those who compete at a higher level, this difference can be interpreted as further proof that controlling unpleasant emotions is a fundamental prerequisite for artistic gymnasts to succeed. Considering that Gold athletes started competing professionally at a very young age, emotion regulation training would, therefore, be particularly appropriate for these athletes. Again in line with H4, significant results for emotional aspects in volleyball players (High− emotions with a positive ß) were consistent with the idea that emotions typically considered unpleasant and dysfunctional (e.g., anger, anxiety) can also be predictive of good performance when they are functional for performance, for example in contact sports (Campo et al., 2012). Open-skills sports can therefore benefit from highly arousing emotions, even if unpleasant. Conversely, closed-skills sports could profit from a lower level of High− emotions for a better control.

Regarding the hypothesis that High− emotions can moderate the relationship between WM and sports performance (H5), we found mixed results. Some research evidence suggested that the relationship between emotions and WM can impact performance. With reference to the two modes of an athlete (Furley et al., 2015), according to reinvestment theory (e.g., Baumeister, 1984; Beilock, 2007), anxiety could burden WM and, therefore, determine “paralysis by analysis” that, in turn, would lead the athlete to massively rely on Type 2 mechanisms (those that require the manipulation of a high cognitive load), even when not needed or even harmful. We did not find a direct moderation effect of high-arousal unpleasant emotions on the relationship between Mcap-Upd and performance in the volleyball players’ sample. A possible explanation was that discrete emotions (e.g., High− emotions) or the hedonic tone dimension alone were not enough to capture the negative moderation effect. Indeed, we found a significant correlation between WM and performance in participants who scored below of the difference between High− and low-arousal of unpleasant emotions and above the mean of pleasant emotions, and in participants who scored above the mean in the difference between High− and low-arousal emotions in both pleasant and unpleasant emotions. These results suggested a combined moderating role of emotions on the relationship between WM and performance. In other words, being happy to compete can buffer the dysfunctional effect of unpleasant emotions (e.g., anxiety) toward performance.

We also explored age and sport differences among youth athletes. According to the literature, 15–17 years-old athletes showed a better performance than younger athletes (11–14 years old) in WM, updating, and inhibition-shifting tasks. This is consistent with both the TCO (Pascual-Leone and Goodman, 1979; Pascual-Leone, 1987), and the developmental framework of EFs suggesting development until early adulthood (Best and Miller, 2010). Moreover, older athletes also tend to adopt an internal attentional focus more than younger athletes, and experience less High− but more Low− emotions prior to competition. This could be linked to older athletes’ better than younger athletes’ inhibitory skills that allow them to better focus on their internal resources and regulate their arousal levels.

The finding that artistic gymnasts performed slightly better on the inhibition-shifting tasks than volleyball players did is in contrast with previous research showing that elite athletes practicing open-skill sports perform better in inhibition (e.g., Krenn et al., 2018). However, this could be due to a generally higher level of sports experience, at the same age, of the artistic gymnasts, as suggested in a recent study (Holfelder et al., 2020). Lastly, compared to artistic gymnasts, volleyball players experienced less High− emotions, and more High− (i.e., excitement) and low-arousal (i.e., calm) pleasant emotions. This is in line with a recent study (Pluhar et al., 2019) reporting team sports athletes to be less likely to suffer anxiety or depression than individual sports athletes. These data could be read from a social perspective and liked to perceived social support (Nixdorf et al., 2016), and diffusion of responsibility (Latané and Darley, 1968). Indeed, team sports athletes tend to benefit from perceived social support more than individual sports ones (Rosenfeld and Richman, 1997). Freeman and Rees (2010) found that the stress buffer generated by social support in team sports is also predictive of better self-confidence at an individual level. Moreover, some researchers suggested that team sports offer to the athlete an opportunity to diffuse responsibility among teammates, minimizing the identifiability of one’s performance, and therefore reducing pre-competitive anxiety (Scanlan and Lewthwaite, 1984; Freeman and Rees, 2010). Thus, pre-competitive anxiety seems helpful for volleyball players’ performance. Moreover, sharing the experience with teammates may evoke happier and less stressful feelings before a competition.

## Conclusion

This research presents some limitations. First, we studied only youth female athletes. Further studies should be carried out considering gender differences, as well as developmental processes, in youth sport. Considering gender as mainstreaming, the integration of a gender perspective in every phase of research development is a priority for sport sciences (Bonato et al., 2022). A second limitation pertains to the measures used to assess predictors. In particular, the measures of inhibition and shifting were not highly correlated, and this could have made the composite measure less reliable. Future research should consider using different EFs measures to better explore the role of EFs in predicting youth sports performance. Moreover, given that our purpose was to measure the emotions generally experienced by athletes prior to a competition, we asked participants to recall how they usually felt before competing. However, since many factors could impact players’ emotions experienced in different competitions, future studies could assess individual emotions prior to several competitive events. Third, a wider sample of participants would allow to systematically test the moderation of emotions on all cognitive predictors.

Despite the limitations, to the best of our knowledge, this is the first investigation that offers an integrated approach to the study of cognitive and emotional predictors in youth sport, considering the differences between open- and closed-skills sports. This research has outlined a clear distinction between the prerequisites necessary for effective performance in an open- and a closed-skills sport: general cognitive skills, such as WM capacity and updating, represent fundamental predictors of performance in volleyball, being a sport in which the athletes need to manage constantly a high load of information. Conversely, in artistic gymnastics, what matters most is the ability of gymnasts to manage pre-competitive anxiety. From a practical point of view, this evidence could result in customized psychological skills training programs tailored to match the sport’s mental requests for youth athletes.

Surprisingly, developmental research has neglected the examination of sports performance [see Kalén et al. (2021)], while sport is an important area for developmental psychology. This research, feeding into a rather small research line (Vestberg et al., 2012, 2017; Verburgh et al., 2014b; Huijgen et al., 2015; Ishihara et al., 2018) provides insights into the relation between cognition, emotions, and performance in youth sport, from a developmental perspective and with an ecological approach to the study of sports performance.

## Data availability statement

The raw data supporting the conclusions of this article will be made available by the authors, without undue reservation.

## Ethics statement

The studies involving human participants were reviewed and approved by University of Verona (Italy) DEDIPAC WP3.2. Written informed consent to participate in this study was provided by the participants or their legal guardian/next of kin.

## Author contributions

EB and FV contributed to the conception and design of the study. EB organized the database and wrote the first draft of the manuscript. EB, FV, and CR performed the statistical analysis. FV, CR, AC, and SR contributed to sections of the manuscript. All authors contributed to the manuscript revision and read and approved the submitted version.
